# Contact Investigation of Multidrug-Resistant Tuberculosis Patients: A Mixed-Methods Study from Myanmar

**DOI:** 10.3390/tropicalmed5010003

**Published:** 2019-12-26

**Authors:** Aye Mon Phyo, Ajay M. V. Kumar, Kyaw Thu Soe, Khine Wut Yee Kyaw, Aung Si Thu, Pyae Phyo Wai, Sandar Aye, Saw Saw, Htet Myet Win Maung, Si Thu Aung

**Affiliations:** 1 TB Department, International Union Against Tuberculosis and Lung Disease (The Union), Mandalay 15021, Myanmar; aungsithu1984@gmail.com (A.S.T.); dr.p.p.wai2012@gmail.com (P.P.W.); sandaraye213@gmail.com (S.A.); 2 Centre for Operational Research, International Union Against Tuberculosis and Lung Disease (The Union), 75006 Paris, France; AKumar@theunion.org (A.M.V.K.); dr.khinewutyeekyaw2015@gmail.com (K.W.Y.K.); 3 Centre for Operational Research, International Union Against Tuberculosis and Lung Disease (The Union), South-East Asia Office, New Delhi 110016, India; 4 Department of Community Medicine, Yenepoya Medical College, Yenepoya (Deemed to be University), Mangaluru 575022, India; 5 Department of Medical Research (Pyin Oo Lwin Branch), Ministry of Health and Sports, Pyin Oo Lwin 05081, Myanmar; kyawthusoe.dmr@gmail.com; 6 Department of Operational Research, International Union against Tuberculosis and Lung Disease (The Union), Mandalay 15021, Myanmar; 7 Department of Medical Research, Ministry of Health and Sports, Yangon 11191, Myanmar; sawsawsu@gmail.com; 8 National Tuberculosis Programme, Ministry of Health and Sports, Nay Pyi Taw 15011, Myanmar; htetmyetwinmaung@gmail.com (H.M.W.M.); sithuaung@mohs.gov.mm (S.T.A.)

**Keywords:** contacts, contact tracing, contact investigation, MDR-TB

## Abstract

There is no published evidence on contact investigation among multidrug-resistant tuberculosis (MDR-TB) patients from Myanmar. We describe the cascade of contact investigation conducted in 27 townships of Myanmar from January 2018 to June 2019 and its implementation challenges. This was a mixed-methods study involving quantitative (cohort analysis of programme data) and qualitative components (thematic analysis of interviews of 8 contacts and 13 health care providers). There were 556 MDR-TB patients and 1908 contacts, of whom 1134 (59%) reached the health centres for screening (chest radiography and symptoms). Of the latter, 344 (30%) had presumptive TB and of them, 186 (54%) were investigated (sputum microscopy or Xpert MTB/RIF^®^). A total of 27 TB patients were diagnosed (six bacteriologically-confirmed including five with rifampicin resistance). The key reasons for not reaching township TB centres included lack of knowledge and lack of risk perception owing to wrong beliefs among contacts, financial constraints related to loss of wages and transportation charges, and inconvenient clinic hours. The reasons for not being investigated included inability to produce sputum, health care providers being unaware of or not agreeing to the investigation protocol, fixed clinic days and times, and charges for investigation. The National Tuberculosis Programme needs to note these findings and take necessary action.

## 1. Introduction

Tuberculosis (TB) is one of the top ten leading causes of deaths in the world. In 2017, there were an estimated 10 million TB patients including 558,000 with resistance to rifampicin (RR-TB), of which 82% had multidrug-resistant TB (MDR-TB, defined as resistance to at least rifampicin and isoniazid) [1]. Myanmar is one of the 30 countries classified as having a ‘high MDR-TB burden’ by the World Health Organization (WHO). There were an estimated 14,000 patients with MDR/RR-TB in 2017 in Myanmar, of whom only 3281 (23%) were reported to be diagnosed by the national TB programme (NTP) [1]. This means that a vast majority of MDR-TB patients remain undiagnosed or not reported to the NTP in Myanmar. 

The “End TB strategy” of the WHO emphasizes early diagnosis and prompt treatment of all TB patients, including drug-resistant TB, to break the chain of transmission and prevent further spread of disease in the community [2]. In line with this, the STOP TB partnership proposes 90-(90)-90 targets (diagnosing and treating 90% of all people with TB, including 90% of the key populations at risk of TB, and achieving 90% treatment success for all people diagnosed with TB) [3]. One such key population group at high risk of TB is ‘household contacts’, living in close contact with source TB patients. Systematic reviews report that the pooled yield of active TB among the contacts is 4.5% [4,5]. A study from New York city reported a yield of active TB of 1% among household contacts investigated [6]. A systematic review among contacts of drug resistant TB patients showed a higher yield of 7.8%, with the majority of secondary cases having the same drug resistance or genotyping pattern as the source case, indicating primary transmission of drug-resistant strains of tuberculosis bacilli [7].

Hence, the WHO recommends ‘contact investigation’—systematic investigation of all household contacts of source TB patients for active and latent tuberculosis and institution of appropriate curative and preventive treatment, respectively [8]. This strategy is endorsed by the NTP in Myanmar and it has been recommended that household contacts of MDR-TB patients with TB symptoms are investigated using Xpert MTB/RIF^®^ assay since 2016 [9].

However, the implementation of this policy is poor and aggregate programme data indicate that contact investigation was done in only 30% of all bacteriologically-confirmed TB patients notified [10]. There is no published evidence about contact investigation among MDR-TB patients from Myanmar, as there is no structured, case-based, recording, reporting, and monitoring of this activity.

The International Union Against Tuberculosis and Lung Disease (The Union), an international non-governmental organization, started implementing a community-based MDR-TB care project in selected townships of Myanmar [11]. As part of this project, community volunteers have been trained and incentivized to conduct many activities including ‘contact investigation’ among MDR-TB patients. This provides an opportunity to assess the extent of implementation of contact investigation, as well as its barriers and possible solutions to address them.

Therefore, we undertook a mixed-methods operational research study with the following objectives: (1) Among the household contacts of MDR-TB patients registered from January 2018 to June 2019, to assess (i) the number and proportion of presumptive TB patients identified, investigated, diagnosed, and treated for TB; (ii) demographic and clinical factors associated with getting or not getting investigated; and (iii) the median duration between the various steps in the cascade. (2) To explore the barriers in implementing contact investigation from the perspective of household contacts and health care providers.

## 2. Methods

### 2.1. Study Design

This was an explanatory mixed-methods study design involving a quantitative component (a cohort analysis of routinely collected programme data) followed by a qualitative component (descriptive study) involving interviews of providers and contacts [12].

### 2.2. Study Setting

#### 2.2.1. General Setting

Myanmar is the second largest country in Southeast Asia, with a population of 52 million people (2014 Census) [13]. About two-thirds of the population lives in rural areas, while the urban populations are concentrated in Yangon and Mandalay regions. Administratively, Myanmar is divided into seven states, seven regions, and one union territory (Nay Pyi Taw), and subdivided into 74 districts with 330 townships [13].

#### 2.2.2. Specific Setting

The study was conducted in 27 selected townships of Mandalay Region, Magway Region, Sagaing Region, and Shan State of Myanmar, implementing the MDR-TB care project with funding support from the Global Fund for AIDS, Tuberculosis, and Malaria. Under this project, the key activities include evening direct observation of treatment for MDR-TB patients, provision of financial incentives to patients, counselling and monitoring of patients for adverse drug effects, health education to family members, and contact investigation. These activities are undertaken by the community volunteers or the project nurses hired and trained for this purpose. Community volunteers are people living in the same ward/village as the patients, but not family members; have reasonable education background (being able to read and write in Myanmar language); have time and interest to learn; and are committed to the care of patients. Peers who have had TB in the past are preferred as volunteers. Community volunteers are supervised by a project focal nurse (one per township) who coordinates with the staff at township level. Every volunteer is assigned a maximum of three MDR-TB patients for providing care. For MDR-TB patients who are not assigned a community volunteer, the project nurse of the respective township conducts the contact investigation, wherever possible.

Both community volunteers and focal nurses receive periodic training on contact investigation and its recording and reporting. The training includes steps to identify household contacts; conduct symptom screening; and refer for investigations, follow-up, and linkage to treatment if required.

#### 2.2.3. Household Contact Investigation

**Figure 1:**
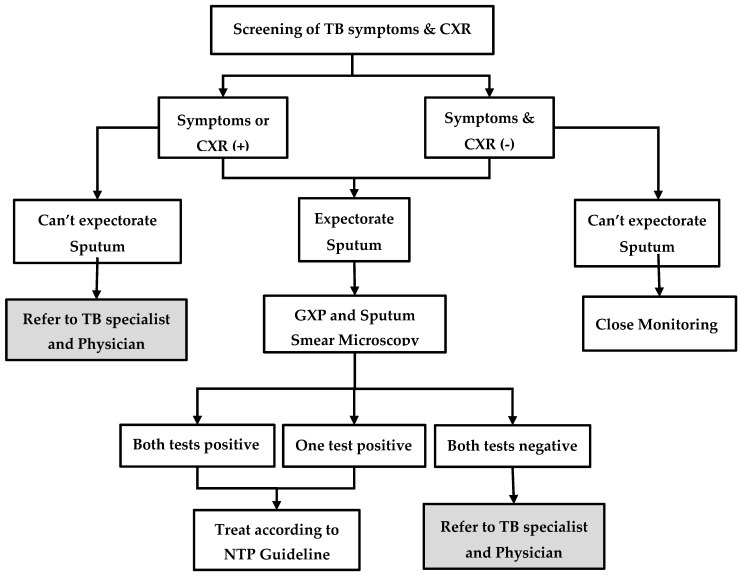
Systematic screening and investigation algorithm for household contacts of index MDR-TB patients in the community-based MDR-TB care project in Myanmar, 2018–19. MDR-TB = multidrug resistant tuberculosis; TB = tuberculosis; CXR = chest X-ray; GXP = Xpert MTB/RIF^®^; TB symptoms = cough, fever, loss of weight, night sweat, and lymph node enlargement.

The process of contact investigation is described in Figure 1. First, the volunteer or the project nurse visits the home of MDR-TB patients and educates them about the importance of contact investigation. Then, the contacts are screened for TB symptoms (cough, fever, weight loss, night sweats, or enlarged lymph nodes) and, regardless of symptoms, they are referred to the township TB centre for chest radiography. If the patient is unable to visit, sputum samples are collected and transported. Irrespective of symptoms or chest radiography findings, people who are able to produce a sputum specimen are investigated further using sputum microscopy for acid-fast bacilli (AFB) and Xpert MTB/RIF^®^ assay. People who are positive for AFB and/or positive for TB bacilli on Xpert MTB/RIF^®^ assay are diagnosed as having TB and are started on first-line or second-line TB treatment, depending on the results of rifampicin resistance. Contacts who are unable to produce sputum or those with ‘negative sputum results, but shadows suggestive of TB on chest radiography’, are referred for further management to the physician, who makes a decision on clinical diagnosis of TB and treatment. In some township TB centres, the facilities for Xpert MTB/RIF^®^ assay and chest radiography are not available. In such situations, contacts are referred to the nearest TB centre or hospital for investigation. 

Household contacts are provided a maximum incentive of 7000 MMK (~5 US$) if they undergo investigation. This is intended to cover the costs of transportation and some investigations like chest radiography, which may not be available free of charge in some townships. 

### 2.3. Recording

The information of index MDR-TB patients including the number of household contacts for each patient is captured in an MS Excel database. There is a “contact register” maintained at the township TB centres, which captures all the details of investigation, TB diagnosis, and treatment of contacts. This information is captured electronically in a quality-assured EpiData database by trained data entry operators and validated periodically by the project supervisors.

### 2.4. Study Population

#### 2.4.1. Quantitative

All household contacts of index MDR-TB patients newly registered in 27 project townships from January 2018 to June 2019 were included. In line with WHO guidelines, a household contact was defined as “a person who shares the same enclosed living space for one or more nights or for frequent or extended periods during the day with the source patient during the treatment or during the three months before commencement of the current treatment”.

#### 2.4.2. Qualitative

The study population includes a purposive sample (maximum variation) of household contacts of MDR-TB patients, community volunteers, and project nurses from selected townships from each region/state. First, we calculated the township-wise Xpert MTB/RIF^®^ testing rates among contacts with presumptive TB. We selected the township with the highest testing coverage and three townships with the lowest testing coverage in such a way that one township was selected from each region/state. In each selected township, two household contacts of MDR-TB patients (one who was investigated and one who was not), two community volunteers, and one project nurse were selected for interviews. In addition, we also interviewed the project supervisor. Thus, a total of 21 interviews were conducted. Participants who were knowledgeable, vocal, and willing to express were purposively selected. The sample size was guided by the saturation of the findings.

### 2.5. Data Variables, Sources of Data, and Data Collection

#### 2.5.1. Quantitative

The data were extracted from electronic databases of the project. The variables included symptoms, chest radiography findings, and results of sputum microscopy and Xpert MTB/RIF^®^ assay. In addition, dates of start of treatment among index patients, contact registration, TB investigation, diagnosis, and treatment start among contacts were collected.

#### 2.5.2. Qualitative

Data collection was done between February and March 2019. Interviews were conducted at a time and place convenient to participants using an interview guide by K.T.S. (a medical doctor from the Department of Medical Research), and K.W.Y.K. (an operational research fellow from The Union), who are trained and experienced in qualitative research (Supplementary File S1) [14]. The guide was pilot tested before implementing in the field. Audio recording was done after receiving consent from participants. Verbatim notes were taken during interview. The average duration of interviews was approximately 45 min. After the interview was over, the summary of the interviews was read back to the participants to ensure participant validation. 

### 2.6. Data Analysis

#### 2.6.1. Quantitative

We analysed using STATA software (version 14.2 STATA Corp., College Station, TX, USA). The demographic and clinical characteristics of the household contacts were summarized using median (inter-quartile range) for continuous variables and frequencies and proportions for categorical variables. The median time between the different stages of the process was calculated.

#### 2.6.2. Operational Definitions

People with either symptoms of TB and/or abnormal shadows on chest radiograph were considered as presumptive TB for this analysis. People who had undergone any of the diagnostic tests (sputum microscopy, Xpert MTB/RIF^®^ assay, or fine needle aspiration cytology) were considered as having been investigated for TB. The date when these investigations were carried out was considered as ‘date of investigation’. If a person underwent more than one investigation, the earlier date was considered. For bacteriologically-confirmed TB patients, the date of the positive test was considered as the date of diagnosis, whereas for clinically diagnosed patients, the date of chest radiography was considered as the date of diagnosis. 

Factors associated with not being investigated for TB and getting tested with Xpert MTB/RIF^®^ assay were measured using adjusted relative risks (RR) and 95% confidence intervals (CI). We initially tried to perform a log-binomial regression. As we did not obtain convergence, a modified Poisson regression with robust error variance was used. Variables that were significant (*p* value < 0.05) in unadjusted analysis or that were known to be associated with the outcome from published literature were included in the multivariable model.

#### 2.6.3. Qualitative

Transcripts were prepared in Myanmar language on the same day of interview based on the audio recordings and verbatim notes. Manual descriptive thematic analysis was performed by the principal investigator [15]. It was reviewed by a second investigator to reduce bias and subjectivity in interpretation. The decision of coding rules and theme generation was done in consensus among investigators. The analysis was done in Burmese language and only the final result was translated into English. The themes are presented for barriers and solutions with the corresponding quotes. Any difference between the investigators was resolved by discussion. We have adhered to the Strengthening the Reporting of Observational Studies in Epidemiology (STROBE) guidelines and ‘Consolidated Criteria for Reporting Qualitative Research (COREQ) in conducting and reporting the study [16,17]. 

### 2.7. Ethics Issues

Ethics approval was obtained from the Ethics Review Committee, Department of Medical Research, Ministry of Health and Sports, Myanmar (Ethics/DMR/2018/159) and the Ethics Advisory Group of The Union, Paris, France (EAG number 48/18). Permission to conduct the study was obtained from the National Tuberculosis Programme, Ministry of Health and Sports, Myanmar. We obtained written informed consent for conducting the interviews and audio recording. A waiver of informed consent was obtained from the ethics committees for quantitative component as this included secondary data analysis.

## 3. Results

### 3.1. Quantitative

**Figure 2:**
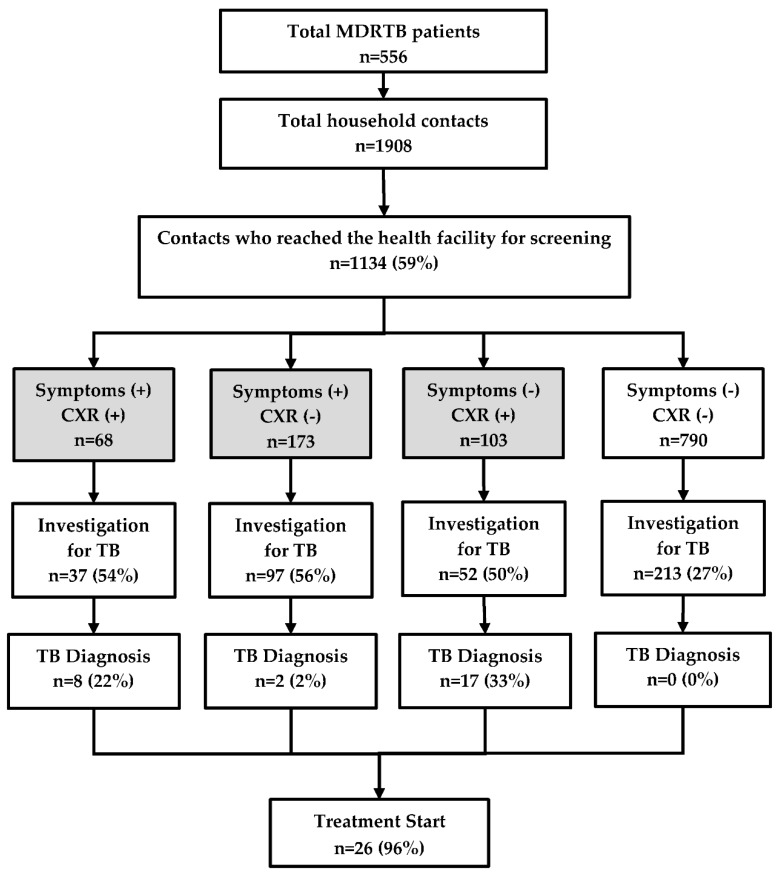
TB investigation, diagnosis, and treatment of household contacts of MDR-TB patients registered in a community based MDR-TB care project in Myanmar, between January 2018 and June 2019. TB = tuberculosis; MDR-TB = multidrug resistant tuberculosis; CXR = chest X-ray. The numbers in the shaded boxes indicate people with presumptive TB defined as those with symptoms (cough, fever, weight loss, and night sweats) and/or abnormal shadows on the chest radiograph.

There were 556 MDR-TB patients who had 1908 contacts living with them. Of the latter, 1134 (59%) reached the township TB centre for screening (Figure 2). The median (inter quartile range, IQR) age of contacts was 30 (14–50) years and 664 (59%) were female.

#### 3.1.1. Cascade of Contact Investigation

Of the 1134 contacts, 344 (30%) had presumptive TB and of them, 186 (54%) were investigated. However, 213 individuals were found to be investigated even though they did not have any symptoms or abnormal chest radiography. Thus, a total of 399 people were investigated for TB and among them, 27 TB patients were diagnosed. Most of the clinically diagnosed cases belonged to the group with ‘no symptoms, but positive findings on chest radiography’ and nearly half of them were children. There was no TB patient diagnosed in the group without TB symptoms that had normal chest radiography. Barring one patient who died, all the remaining 26 (96%) patients started on the treatment (Figure 2).

The characteristics of TB patients are shown in Table 1. Of the 27 patients, six had bacteriologically-confirmed TB, while the rest were clinically diagnosed. Of the six bacteriologically-confirmed, five had pulmonary TB (all with rifampicin resistance) and one had extrapulmonary TB who was AFB-positive on Fine Needle Aspiration Cytology aspirate. All were new cases, barring one who reported a previous history of TB. A total of 362 contacts underwent sputum microscopy and only one was AFB-positive. Xpert MTB/RIF^®^ assay was conducted among 176 contacts and 5 were diagnosed as TB (this included the one case diagnosed by sputum microscopy).

#### 3.1.2. Factors Associated with Not Being Investigated for TB

Of 344 presumptive TB patients, 158 (46%) were not investigated for TB. In adjusted analysis, failure to do TB investigation was significantly higher among the contacts who were less than 15 years old, those who were registered in health facilities without an Xpert MTB/RIF^®^ machine, and those who were referred when compared with those whose sputum was collected and transported to health facilities by project staff (Table 2).

#### 3.1.3. Factors Associated with Getting Tested for Xpert MTB/RIF^®^

Of 344 presumptive TB patients, 121 (35%) were tested using Xpert MTB/RIF^®^. In the adjusted analysis, the Xpert MTB/RIF^®^ testing was significantly lower among contacts aged less than 15 years and significantly higher in health facilities with an Xpert MTB/RIF^®^ machine on-site (Table 3).

#### 3.1.4. Delays

The median (IQR) duration between treatment start of index case to contact screening at the township TB centre was 81 (28–208) days. Among those investigated, 75% underwent the investigation within a day. The median time to treatment from diagnosis was 8 days—this was 14 days among bacteriologically-confirmed patients, but 4 days among clinically diagnosed patients (Table 4).

### 3.2. Qualitative

Implementation barriers in contact investigation were multi-factorial and inter-related with each other. We organized the barriers under two broad themes—household contacts-related barriers and health system-related barriers. The barriers summarized here reflect the perspectives of both the household contacts and health care providers. Overall, the participants from the townships with low testing coverage reported a greater number of barriers and, more predominantly, health system barriers. The verbatim quotes (translated in English) are italicized and placed within double quotes.

#### 3.2.1. Household Contact-Related Barriers

##### Unable to Visit the Clinic

Working people and school-going children were unable to visit the township TB centre for investigation because the clinic times conflicted with the work/school timings.

“Some contacts were students. So they have to attend school from Monday to Friday. They can’t come on these days for taking CXR (Chest X ray).”

*(Community volunteer-5)*

“Contacts did not want to go to OPD (Outpatient Department) because they didn’t want to absent their jobs.”

*(Project Nurse-3)*

The other barrier was related to distance requiring a long time to travel, which was compounded by personal problems.

“Some contacts couldn’t come because they were very old and they lived far away”

*(Project Nurse-1)*

“I feel motion sickness when I travel… Therefore, I rarely travel”

*(tested household contact-1)*

##### Inability to Produce Sputum

Some contacts could not produce sputum at all or only an inadequate amount of sputum for investigation.

“Sayarma (The Nurse) gave the sputum cup to me and told to produce sputum. But I can’t produce the sputum.”

*(Non-tested Household contacts-4)*

##### Financial Constraints

Some contacts, especially daily wage labourers, were reported to have financial constraints associated with visiting the township TB centre, as it meant absence from work and loss of daily wages, in addition to transportation charges. Although the project supported their travel allowance, it was a fixed amount and did not cover all the expenses.

“They could not spend time for investigation. They are daily-wages workers. Therefore, they need to work for their daily income.”

*(Project Nurse-4)*

“For the contacts who lived far away from township TB centre, there are higher transportation costs. Although project supports this cost, it is not enough for them.”

*(Project Nurse-3)*

##### Beliefs and Attitude

Some contacts refused to do contact investigation because they did not have any signs and symptoms and strongly believed that they do not have the disease. Others did not want to undergo investigation because they were afraid of possible side-effects of the TB drugs in the eventuality that they were diagnosed to have TB. One person mentioned that *God will take care of her illness*, even if it existed.

“Contacts said that they believed that they have no disease (TB). So they don’t want to test.”

*(Community volunteer-8)*

“I heard TB patients are afraid of the injections and they can’t withstand the side-effects, so do I.”

*(Not-tested household contacts-3)*

#### 3.2.2. Health System-Related Barriers

##### Lack of or Inadequate Counselling

Not all MDR-TB patients were assigned a volunteer in the project. So, the contact investigation may not have been done in such patients. The project nurse reported that some volunteers were not able to communicate and counsel effectively and convince the contacts to undergo TB screening.

“Volunteers could not explain well about the importance of TB screening to contacts”

*(Project Nurse-2)*

“No one told me how to produce sputum”

*(non-tested contact-4)*

The project supervisor reported that some of the contacts had already been investigated by the time volunteer visited the home, and hence were not referred. Such contacts were not recorded in the project database.

##### Do Not Know

Some of the health care providers at the township TB centre were not aware of the contact investigation protocol. So, chest radiography was not provided for asymptomatic contacts.

“Even if the contacts reached the health facility, health care providers at TB centre did not offer chest X ray, because they had no signs and symptom of TB”

*(Project Nurse-3)*

##### Do Not Agree

Some of the health care providers were aware of the protocol followed in the project, but did not agree, because it did not align with the NTP guidelines. While the project protocol advocated for screening using chest radiography in addition to symptom screening, NTP recommends only symptom screening and further investigation is limited to those with symptoms.

“The TB focal person informed us that if there are no symptoms, we cannot do any investigation”

*(Project nurse-3)*

##### They Do Not Do: High Workload

It was reported that investigations were not offered to contacts by the staff of the TB centre for various reasons. One was related to the high workload and shortage of human resources in laboratory unit in the township TB centre. Some of the staff at the township TB centre were unable to pay attention to the contact investigation as they were engaged with multiple responsibilities.

“The laboratory technician position is vacant in TB centre”

*(Project Nurse-2)*

“The focal person does not involve fully in TB related activity as he also worked for other public health programmes. He is always busy”

*(Project Nurse-4)*

##### They Do, But on Fixed Days and Times

Some laboratories had a fixed time to receive sputum specimens from the patients. If the patients arrived outside the times, they were asked to return the next day. Sputum specimens received outside the fixed times were discarded and this meant requesting contacts for additional specimens. In some health facilities, the chest X-ray unit imposed restrictions on the number of chest radiographs that could be taken on a given day (such as a maximum of 10 persons per day). All of the others were asked to come on the next day. This was very inconvenient for the contacts who had travelled from far off places. Similarly, there was a fixed day in a week for doctors to examine presumptive TB patients and make a decision about clinical diagnosis.

“The laboratory accepts sputum sample between 9 am and 10 am only. Specimens received outside this time are discarded and then it is difficult to request for additional specimens from contacts.”

*(Project Nurse-4)*

“Chest X ray unit opens at 9 am and they allow only 10 persons per day to take chest X ray from TB department. Therefore, when the contact came and if it is beyond their maximum number, this person is asked to return the next day. And, the contact may not return.”

*(Project Nurse-1)*

##### They Do, But They Charge

It was reported that the contacts had to pay to undergo chest radiography in some places.

“Chest X-ray fee is high. Here, it is 1500 MMK and this charge is higher in other township hospitals.”

*(Project Nurse-3)*

## 4. Discussion

This is the first study from Myanmar providing information on contact investigation among MDR-TB patients and its implementation challenges. We discuss the magnitude of gaps at each step of the cascade and their reasons below.

One of the main gaps was that nearly four in ten contacts did not reach the health facility for screening. This is higher than that reported from South Africa and similar to Ethiopia [18,19]. The possible reasons included lack of knowledge about the need for contact investigation, lack of risk perception owing to wrong beliefs, financial problems related to loss of wages and high transportation charges not entirely reimbursed by the project, and conflicts of clinic times with work/school times. It is possible that home visits and educating about contact investigation may not have happened in some MDR-TB patients. An interesting observation revealed during key informant interviews was that several contacts had already been investigated for TB by the time the project staff made home visits. Such people were not included in the numerator, but were counted in the denominator, when calculating this indicator, thus marginally overestimating the proportion not reached.

The next gap was at the level of screening and investigating the contacts who had reached the health facility. Only half of the presumptive TB patients received any investigation for bacteriological confirmation. The children were less likely to be tested, mostly because they were unable to produce sputum, and the gastric lavage was not routinely done in our setting, which requires hospitalization. Access to health facilities was another factor. The contacts who lived in townships that had Xpert MTB/RIF^®^ facility were more likely to be tested as it reduced the travel cost and time. 

Contacts whose sputum samples were collected at home and transported by project staffs were more likely to be investigated than contacts who had reached the health facility. Because investigation required two sputum samples, as per NTP guidelines, contacts referred to health facilities had to make multiple visits. Sometimes, the nearest health facility (to where the contacts were referred) did not have Xpert MTB/RIF assay services. In such instances, contacts had to be referred to another health facility with Xpert services. All these were reported as inconvenient and may have led to the losses in the cascade. While some TB focal persons at the township TB centre were unaware about the contact investigation protocol, some disagreed with the requirement of screening all contacts with chest radiography. There was also confusion among the providers about the eligibility criteria for prescribing the Xpert MTB/RIF^®^ assay. The other barriers included fixed times and days for receiving sample or patients and demanding charges for investigations.

This study had several strengths and some limitations. First, we included a large sample of contacts covering 27 project townships of four states and regions. Thus, the findings are likely to be representative of the situation in these areas. Second, we used a mixed-methods study design, which helped in understanding the underlying reasons for the gaps in care cascade. Third, we used quality-assured data collected by project staff, which is routinely monitored and validated. Fourth, we achieved saturation in our qualitative interviews. Fifth, we followed the STROBE and COREQ guidelines for reporting the quantitative and qualitative components, respectively [16,17]. One limitation was that we had no information on 40% of household contacts who did not reach the health facility for screening; hence, we do not know if they were similar to those who reached the health facility. The impact of this on overall findings is unclear. Another limitation was that we did not interview the health care providers responsible for providing TB services and include their perspectives. This should be considered in future research.

Despite this limitation, our findings have many implications for programme policy and practice. First, we recommend that chest radiography be used for screening all household contacts regardless of TB symptoms, wherever possible, because the yield of TB was highest in the group with ‘no symptoms, but abnormal chest radiograph’. Although most of the cases in this group were clinically diagnosed, there was one case of rifampicin resistance too. We may have diagnosed more cases of TB, had we tested everyone with Xpert MTB/RIF^®^ assay. However, the feasibility of this recommendation needs to be tested before wider scale-up.

Second, we recommend that contacts ‘without TB symptoms and normal chest radiograph’ should not be investigated any further because there was zero TB in this group. This is also supported by evidence from systematic reviews [19]. A substantial number of patients were unnecessarily investigated and the resources could have been used elsewhere to increase the testing rates among presumptive TB patients. 

Third, as shown in our study, the prevalence of drug resistant TB among contacts of MDR-TB patients is high in studies conducted elsewhere [7,20,21,22]. Hence, Xpert MTB/RIF^®^ test should be the first diagnostic test of choice, as recommended by WHO [23]. There was no additional yield of TB owing to sputum microscopy in our study. Hence, we recommend discontinuing sputum microscopy for contacts of MDR-TB patients and focusing on the Xpert MTB/RIF^®^ assay, as it can reduce the workload at township laboratories. This strategy can also be more convenient for the contacts, because sputum microscopy requires two specimens requiring multiple visits, whereas Xpert MTB/RIF^®^ testing requires only one specimen.

Fourth, refresher training should be conducted periodically for community volunteers to improve their knowledge about contact investigation and counselling skills. The training content can be tailored to resolve the specific myths and beliefs among the contacts. 

Fifth, efforts should be made to bring the contact investigation services closer to the community. This includes strengthening of sputum collection and transportation to township TB centres. However, this alone will not obviate the need for visiting health facility, as contacts also have to undergo chest radiography. To address this, we recommend exploring the possibility of using new technologies such as digital chest radiography with automated computer-aided detection of tuberculosis, which can be mounted in a mobile van for greater outreach [24]. 

In conclusion, we identified the magnitude of gaps in the cascade of contact investigation among MDR-TB patients in Myanmar, as well as reasons for the same. We hope these findings can be shaped into practical recommendations that will inform the NTP in Myanmar.

## Figures and Tables

**Table 1:** Characteristics of TB patients diagnosed among household contacts of MDR-TB patients registered in a community based MDR-TB care project in Myanmar, between January 2018 and June 2019.

Characteristics	TB Patients	
	N	(%)
Total	27	(100)
**Age group (years)**		
≤14	10	(37.0)
15–44	12	(44.4)
45–64	4	(14.8)
≥65	1	(3.70)
**Sex**		
Male	12	(44.4)
Female	15	(55.6)
**Rifampicin resistance**		
Not tested	7	(25.9)
No	15	(55.6)
Yes	5	(18.5)
**Type of TB**		
Bacteriologically-confirmed	6	(22.2)
Clinically diagnosed	21	(77.8)
**Site of TB**		
Pulmonary TB	26	(96.3)
Extrapulmonary TB	1	(3.7)

TB = tuberculosis; MDR-TB = multidrug resistant tuberculosis.

**Table 2:** Factors associated with not being investigated for TB among household contacts with presumptive TB registered in a community-based MDR-TB care project in Myanmar, between January 2018 and June 2019.

Characteristics	Total	Not Investigated		RR	(95%CI)	aRR	(95%CI)
	N	N	(%)				
Total	344	158	(45.9)				
**Age (years)**							
≤14	93	62	(66.7)	1.77	(1.37–2.28) *	1.47	(1.15–1.89) *
15–44	143	54	(37.8)	Ref		Ref	
45–64	82	34	(41.5)	1.10	(0.79–1.53)	1.13	(0.83–1.53)
≥65	26	8	(30.8)	0.81	(0.44–1.51)	0.90	(0.52–1.58)
**Gender**							
Male	145	64	(44.1)	Ref		Ref	
Female	199	94	(47.2)	1.07	(0.85–1.35)	1.14	(0.92–1.42)
**Cough**							
Yes	185	71	(38.4)	Ref		Ref	
No	159	87	(54.7)	1.43	(1.13–1.80) *	1.08	(0.87–1.34)
**Fever**							
Yes	27	17	(63)	1.42	(1.03–1.94) *	NE	
No	317	141	(44.5)	Ref			
**Loss of weight**							
Yes	65	35	(53.8)	1.22	(0.94–1.59)	NE	
No	279	123	(44.1)	Ref			
**Health Facility**							
Without GXP	40	28	(70)	1.64	(1.29–2.08) *	1.60	(1.24–2.07) *
With GXP	304	130	(42.8)	Ref		Ref	
**Refer type**							
Patient	299	157	(52.5)	23.63	(3.39–164.6) *	20.46	(2.88–145.53) *
Sputum Sample	45	1	(2.2)	Ref		Ref	
**State/Region**							
Mandalay	196	83	(42.3)	Ref		Ref	
Sagaing	76	37	(48.7)	1.15	(0.87–1.53)	1.20	(0.89–1.62)
Shan	38	19	(50)	1.18	(0.83–1.69)	1.18	(0.85–1.65)
Magway	34	19	(55.9)	1.32	(0.94–1.85)	1.06	(0.74–1.51)

TB = tuberculosis; MDR-TB = multidrug resistant tuberculosis; GXP = Xpert MTB/RIF^®^ machine; CI = confidence interval; RR = relative risk; aRR = adjusted relative risk; *n* = number; NE = not estimated.* = statistically significant. The variables that were significant in the unadjusted analysis and that were found to be associated in previous studies were included in the adjusted analysis. Fever was not included in the adjusted model owing to collinearity with cough.

**Table 3:** Factors associated with GXP testing among household contacts with presumptive TB registered in a community-based MDR-TB care project in Myanmar, between January 2018 and June 2019.

Characteristics	Total	GXP Tested		RR	(95%CI)	aRR	(95%CI)
	N	N	(%)				
Total	344	121	(35.2)				
**Age (Years)**							
≤14	93	22	(23.7)	0.53	(0.35–0.79) *	0.54	(0.35–0.82) *
15–44	143	64	(44.8)	Ref		Ref	
45–64	82	28	(34.1)	0.76	(0.54–1.08)	0.75	(0.53–1.05)
≥65	26	7	(26.9)	0.60	(0.31–1.16)	0.59	(0.31–1.12)
**Gender**							
Male	145	53	(36.6)	Ref		Ref	
Female	199	68	(34.2)	0.93	(0.70–1.25)	0.95	(0.72–1.25)
**Cough**							
Yes	185	75	(40.5)	1.40	(1.04–1.89) *	1.06	(0.78–1.45)
No	159	46	(28.9)	Ref		Ref	
**Health Facility**							
Without GXP	40	7	(17.5)	Ref		Ref	
With GXP	304	114	(37.5)	2.14	(1.08–4.27) *	2.14	(1.1–4.17)
**Refer type**							
Patient	299	101	(33.8)	Ref		Ref	
Sputum Sample	45	20	(44.4)	1.32	(0.92–1.89)	1.16	(0.8–1.69)
**State/Region**							
Mandalay	196	76	(38.8)	Ref		Ref	
Sagaing	76	21	(27.6)	0.71	(0.48–1.07)	0.67	(0.44–1.03)
Shan	38	18	(47.4)	1.22	(0.84–1.78)	1.21	(0.85–1.73)
Magway	34	6	(17.6)	0.46	(0.22–0.96) *	0.51	(0.24–1.08)
**Symptoms**							
No Symptom	103	31	(30.1)	Ref		NE	
Any Symptom	241	90	(37.3)	1.24	(0.89–1.74)		

TB = tuberculosis; MDR-TB = multidrug resistant tuberculosis; GXP = Xpert MTB/RIF^®^ machine; CI = confidence interval; RR = relative risk; aRR = adjusted relative risk, *n* = number; NE = not estimated. * = statistically significant. The variables that were significant in the unadjusted analysis and that were found to be associated in previous studies were included in the adjusted analysis.

**Table 4:** Median duration (days) between different steps in the cascade of contact investigation among household contacts registered in community-based MDR-TB care project in Myanmar, between January 2018 and June 2019.

Duration (Days)	Total Eligible	Number (%) with Valid Dates	Median Days	(IQR)
Treatment start of index MDR-TB case and contact screening	1134	1005 (89)	81	(28–208)
Contact screening and investigation	399	380 (95)	0	(0–1)
TB diagnosis and treatment initiation	26	26 (100)	8	(2–14)
Bacteriologically-confirmed TB	5	5 (100)	14	(14–15)
Clinically diagnosed TB	21	21 (100)	4	(2–10)

TB = tuberculosis; MDR-TB = multidrug resistant tuberculosis; IQR = inter quartile range.

## Supplementary Materials

The following are available online at https://www.mdpi.com/2414-6366/5/1/3/s1, S1: Interview Guide for Key Informant Interview.
