# Supplementary material for: Contact Investigation of Multidrug-Resistant Tuberculosis Patients: A Mixed-Methods Study from Myanmar
Source: Trop Med Infect Dis. 2019 Dec 26;5(1):3. doi: 10.3390/tropicalmed5010003 (PMC7157597; doi:10.3390/tropicalmed5010003)
Supplement: Supplementary file 1 [file tropicalmed-05-00003-s001.pdf]

Annex 1: In-depth Interview: Interview guide for household contacts of MDR-TB patient

Name of the participant: **Designation:**

Date of Interview: **Interview start / end time:**

Name of the Interviewer:

After a brief introduction to the participant regarding the purpose of the interview, the PI will obtain written informed consent for the interview. Written informed consent will also be obtained for audio recording

1. What are your views about the risk of TB and MDR-TB infection?
2. According to you, what are the infection control measures of MDR-TB among healthy people? [Probe: healthcare provider level, patient level]
3. Have you known or heard about contact investigation activity (TB examination for those who closely stay with MDR-TB patients) by healthcare provider? [Probe: programmatic level, patient level]
4. Have you been investigated for TB before? If yes, probe for details. If no, probe for reasons.
5. What do you think are the benefits of contact investigation? [Probe: programmatic level, patient level]
6. What do you think are the disadvantages contact investigation? Probe: programmatic level, patient level]
7. What is your opinion regarding the contact investigation implementation? [Probe: Programmatic level, Patient level]
8. What are your challenges to undergo contact investigation? [Probe: Programmatic level, Patient level]
9. Is there any suggestion to solve the problems of contact investigation? [Probe: programmatic level, patient level]
10. Additional remarks, if any?

PI will complete the interview by acknowledging the time spared by the participant from his/her busy schedule. She will also give a summary of the notes taken and confirm the same from the participant.

Annex 2: In-depth Interview: Interview guide for healthcare provider

Name of the participant: **Designation:**

Date of Interview: **Interview start / end time:**

Name of the Interviewer:

After a brief introduction to the participant regarding the purpose of the interview, the PI will obtain written informed consent for the interview. Written informed consent will also be obtained for audio recording

1. Have you received training or/and guidelines to do contact investigation? [Probe: Programmatic level]
2. How do you identify the household contacts of MDR-TB?
3. How do you perform contact investigation? [Probe: giving refer form only, giving refer form and facilitate to receive the results of investigation]
4. What do you think about the feasibility of implementing contact investigation in routine settings? [Probe: programmatic level – How can it be implemented on large scale]
5. What are the benefits of contact investigation?
6. What do you think are the challenges in contact investigation implementation? [Probe: programmatic level, patient-level]
7. What is your opinion regarding the acceptability of systematic contact investigation implementation [Probe: programmatic level, patient-level]
8. What are the major operational issues in implementation? [Probe: both at provider level and patient level]
9. What is your suggestion to improve contact investigation implementation? [Probe: programmatic level, patient-level]
10. Additional remarks, if any?

PI will complete the interview by acknowledging the time spared by the participant from his/her busy schedule. She will also give a summary of the notes taken and confirm the same from the participant.
